# An evolutionary perspective on the use of betel nut and its effects on health outcomes

**DOI:** 10.1093/emph/eoaf037

**Published:** 2025-12-15

**Authors:** Laura Perez, Katherine Wander, Kristin K Sznajder, Nurul Alam, Rubhana Raqib, Farjana Haque, Anjan Kumar, Tami Blumenfield, Siobhán M Cully, Mary K Shenk

**Affiliations:** College of Medicine, Pennsylvania State University, 700 HMC Crescent Road Hershey, PA 17033, USA; Department of Anthropology, Pennsylvania State University, 237 Susan Welch Liberal Arts Building, University Park, PA 16802, USA; Department of Anthropology, Binghamton University, State University of New York, 4400 Vestal Parkway East, Binghamton, NY 13902, USA; Department of Public Health Sciences, Pennsylvania State University, Hershey, PA, USA; icddr,b, 68 Shaheed Tajuddin Ahmed Ave, Dhaka 1212, Bangladesh; icddr,b, 68 Shaheed Tajuddin Ahmed Ave, Dhaka 1212, Bangladesh; icddr,b, 68 Shaheed Tajuddin Ahmed Ave, Dhaka 1212, Bangladesh; icddr,b, 68 Shaheed Tajuddin Ahmed Ave, Dhaka 1212, Bangladesh; Department of Anthropology, University of New Mexico, Albuquerque, NM, USA; Department of Anthropology, University of New Mexico, Albuquerque, NM, USA; Department of Anthropology, Rutgers University, New Brunswick, NJ, USA; Department of Anthropology, Pennsylvania State University, 237 Susan Welch Liberal Arts Building, University Park, PA 16802, USA

**Keywords:** psychoactive substances, evolutionary tradeoffs, health outcomes, betel nut, Bangladesh

## Abstract

**Background and objectives:**

The use of psychoactive substances appears to be a consistent behavior throughout human evolutionary history. In contemporary research, this is often attributed to the addictive properties of such substances; an evolutionary perspective offers a more nuanced view. We take the case of betel nut use in Bangladesh to investigate the relationship between betel quid and chronic health outcomes, and to consider local disease ecology and evolutionary explanations for consumption of this psychoactive substance.

**Methodology:**

We analyzed data from a random sample of 765 women and 499 men in Matlab, Bangladesh, to assess associations between betel quid use and anemia, type 2 diabetes (T2D), hypertension, and inflammation (C-reactive protein, CRP).

**Results:**

Betel quid use was associated with all health outcomes investigated. Use of betel quid was inversely associated with CRP (β = −0.34; *P*-value = .007). For other outcomes, there were important interactions between betel quid use and gender. A positive association with anemia (aOR: 2.56, CI: 1.62, 4.04) and inverse associations with diabetes and hypertension (aOR: 0.38, CI: 0.22, 0.66; aOR: 0.41, 1.03, respectively) were apparent among men, but not women (anemia: aOR: 1.03, CI: 0.72, 1.49; diabetes: aOR: 0.98, CI: 0.58, 1.65; hypertension: aOR: 1.25, CI: 0.85, 1.85).

**Conclusions and implications:**

Betel quid use was inversely associated with inflammation and, among men, positively associated with anemia and inversely associated with diabetes and hypertension. Together, these findings suggest that the use of betel quid, and possibly other addictive substances, may have been a behavioral adaptation to diverse socioecological challenges.

## BACKGROUND AND OBJECTIVES

Betel nut, consumed by ~10% of the world’s population, is the fourth most used psychoactive substance after nicotine, alcohol, and caffeine [1]. Betel nut is typically consumed as “betel quid”, a combination of the ripe seed of the *Areca catechu* palm (betel nut), the leaf of the *Piper betle* (betel leaf), and occasionally tobacco and spices [1]. Betel quid consumption is most common in Asia and Oceania, with evidence of its use dating back to 400 BCE [2–5]. Reported reasons for use include its stimulant properties, relief of gastrointestinal ailments, and anthelminthic properties [6]. However, studies link its use with type 2 diabetes (T2D), hypertension, and oral cancer [7]. This contrast between traditional, often medicinal, use of betel quid and biomedical perspectives is echoed in research on many psychoactive substances [8–10].

Psychoactive substance use is a widespread, cross-cultural practice throughout human history [11, 12], often included in ritualistic or medicinal practices [13]. The relatively recent capacity to refine and concentrate psychoactive substances has contributed to the global rise of substance use disorders, prompting evolutionary hypotheses that investigate the human propensity for addiction [14]. A second line of inquiry around traditional use of psychoactive compounds—understanding why humans consume them in the first place—has received less attention but is no less important. Psychoactive substances often have medicinal properties or contain pharmacotherapeutic compounds; our affinity for these substances may be due to the medicinal benefits associated with their use [15–20].

### Self-medication in non-human primates

Emerging evidence shows self-medication behaviors extend beyond humans, for example, non-human primates have been observed consuming or using plants beyond their typical diet, potentially for their therapeutic properties. Two famously psychoactive plants, *Cannabis sativa* and *Tabernanthe iboga,* are consumed by Japanese macaques and Western and Eastern lowland gorillas, respectively. Among human populations, *C. sativa* has been used for fatigue, pain, and nausea, and pharmacological studies have attributed these properties to the plant’s active metabolites (e.g. tetrahydrocannabinol, terpenoids, alkaloids, etc.). Ibogaine, a compound in *T. iboga*, has situated the plant at the center of spiritual and ethnomedicinal practices in West-Central Africa [15]. Beyond its hallucinogenic potential, iboga has been used for fatigue and depression, and has been shown to have anti-parasitic properties [15, 21]. Time-restricted use of these plants by animals during seasons or periods of high risk of infection/illness supports the interpretation that this use is medicinal or therapeutic in nature [22], suggesting that consuming plants with psychoactive properties may be an adaptive response to dynamic disease ecologies.

### Archeological context

Despite limited preservation of plants and fungi in the archeological record, and uncertainty about how such species were used [23], there are clear indications of use of psychoactive or medicinal substances deep in the past. For example, *Ephedra*, a plant genus that includes *Ephedra sinica*, from which ephedrine was isolated, has been found among *Homo neanderthalensis* remains in multiple sites [24–26]. Similarly, recent analyses of DNA preserved in dental calculus of a Neanderthal individual from northern Spain identified a gastrointestinal parasite, pathogenic oral microbes, and a species of poplar (*Populus trichocarpa)* which contains salicylic acid (the active ingredient in aspirin) [27].

### Modern cross-cultural context

**Figure 1:**
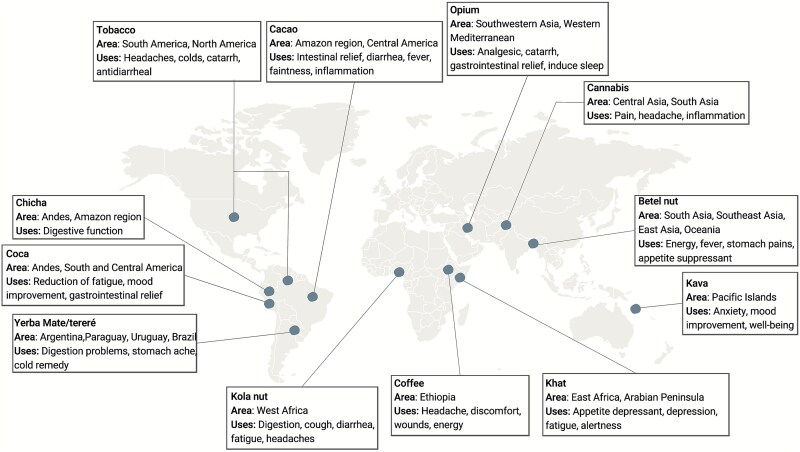
Representative psychoactive substances, including their proposed regions of origin and reported traditional medicinal properties.

Psychoactive substances have been integral to ethno-medicinal traditions worldwide (Fig. 1). In the Americas, tobacco, coca, cacao, and the components of chicha and Yerba Mate have long been used for ritual and health purposes [8]. Tobacco is reported to alleviate headaches, colds, and postnasal drip [28]. Coca has been traditionally used for fatigue, mood enhancement, and gastrointestinal relief, and remains an important part of Andean culture today [29]. Cacao, whose use dates back ~5300 years, has been implemented for treatment of fevers and diarrhea [30, 31]. Chicha (i.e. fermented maize, yuca, or quinoa) is said to enhance digestive function [32]; some biomedical research attributes its effects to the probiotics that arise during the fermentation process [33]. Indigenous communities in Ecuador report using chicha and tobacco leaves as a treatment for chills and fever in cases of malaria [34]. Yerba mate (traditionally, *Ilex paraguariensis)* has been used with other medicinal plants to treat stomachaches, colds, and sexually transmitted infections [35].

In Asia, opium and cannabis have a long-recorded history of use primarily for their analgesic properties [36–38]. Like coca, derivative forms of opium have become substances of abuse but are also regularly used as painkillers within biomedicine [39–41].

Kava, native to the Pacific islands, has been domesticated for thousands of years and used for anxiety, mood improvement, and pain relief [42]. Kava has been found to reduce spasms, muscle contractions, and involuntary movements among patients with a psychiatric diagnosis who were treated with antipsychotic medications [43, 44].

In West Africa, kola nut has historically been chewed for colic, both dysentery and constipation, coughs, and other ailments [9, 45]. In East Africa, khat has traditionally been chewed for its stimulant properties, while coffee and coffee grounds have been used for headaches and wound dressings, respectively [10, 46].

Despite these reported—and sometimes substantiated—medicinal properties, a significant portion of the available biomedical research on psychoactive substances is focused on adverse health outcomes associated with their use. For example, much of the literature on khat investigates risk for cardiovascular events and periodontal damage [47, 48]. In the case of kola nut and Yerba Mate, there are warnings about their possible association with oral and esophageal cancers, while raw opium has been associated with increased risk of cardiovascular disease [44, 49, 50].

The domestication of a plant with minimal nutritional value seems unlikely for its addictive properties alone. The many examples of such domesticates, with long histories and persistent use prior to any modern refinement, suggest health benefits may exist and should be examined in the context of local disease ecologies.

### The case of betel quid

Betel quid is an ideal case study for understanding psychoactive substance use: medicinal use is reported, yet biomedical research suggests adverse health effects [51]. Traditionally, betel nut has been used as an anthelmintic and to treat fever, sexually transmitted infections, stomach pain, and rheumatism [52]. Betel quid users may also have milder schizophrenic symptoms than non-users [53, 54] and generally report improved sense of well-being, increased energy, and improved digestion [55]. These effects may be in part attributed to the actions of betel nut’s active compound, arecoline, on the central nervous system and parasympathetic nervous system [56].

Nonetheless, the overwhelming majority of available studies report adverse physiological effects of betel quid use [51]. Betel quid has been associated with increased risk of cardiovascular disease, hypertension [7, 57], and T2D, although one study found no association with T2D, and some animal studies suggest a protective effect from arecoline [58–61].

We tested the associations between betel quid use and four chronic health outcomes (two of which were previously explored in standalone papers and replicated here, along with two novel health outcomes) among a cross-sectional sample of men and women living in Matlab *upazila* [62, 63]*.* Given our previous findings and what is known about betel quid use, we hypothesized that protective effects of betel quid were more likely for the diseases that have a long history in this setting, while adverse effects were more likely for diseases that have only recently become more prevalent (i.e. those associated with nutrition transitions and market integration) [64].

## MATERIALS AND METHODS

### Research setting

We used data collected as part of a larger independent study on demography, health, and market integration in Matlab, Bangladesh, in 2018, in association with icddr,b (International Centre for Diarrheal Disease Research, Bangladesh). Bangladesh has been experiencing an epidemiologic transition from communicable diseases to non-communicable diseases, concurrent with a nutritional transition and increasing market integration [65]. Rates of chronic disease have been increasing both in urban and rural settings [65, 66]. Betel quid is widely used by both men and women in Bangladesh, with most frequent use among men and those of lower socioeconomic status (SES) in rural areas [67, 68].

### Study design and participants

Participants were initially randomly selected in 2010 within 15-year age strata from a complete population roster curated by icddr,b as part of a longstanding Health and Demographic Surveillance System (HDSS) site [69]. Women were recruited and were eligible to participate if they were between the ages of 20 and 65 in 2010. The 2018 data collection expanded eligibility to men married to participating women who were present in Matlab at the time of data collection. The data reported here were collected in 2018 from 765 women (2010 participants who in 2018 were still alive, not pregnant, and residing in the study area) and 499 men (many husbands were labor migrants living outside the study area, and some husbands had died or been divorced from their wives). Attrition after the 2010 study was mainly due to migration (Table S1).

### Survey

Surveys were piloted by co-authors Shenk and Alam and then implemented by trained icddr,b field staff in Matlab. The data in the surveys included individual and household information, age, outcome measures of interest, betel quid use frequency, and sociodemographic variables including: MacArthur Scale of Subjective Social Status (a measure of SES), years of education, food security (always or not always having enough quality food), food source (all, some, or no food regularly purchased from the bazaar), occupation for men, tobacco use for men, secondhand exposure to tobacco smoke for women (women rarely smoke in this context), and whether the toilet was owned or shared (as a proxy for exposure to infectious disease).

### Specimen collection and outcome measurements

Blood pressures were measured for each participant using a digital sphygmomanometer (Omron HEM-705CP). Digital capillary whole blood was collected via finger stick and immediately evaluated for glycated hemoglobin (HbA_1c_; A1C Now, PTS Diagnostics) and hemoglobin (Hb; HemoCue 201+). Additional drops of whole blood were collected on filter paper (Whatman #903) for dried blood spots (DBS), dried for up to 24 hours, and stored at −20°C until analysis. DBS were evaluated in the Immunobiology, Nutrition, and Toxicology Laboratory at icddr,b for C-reactive protein (CRP), a biomarker of inflammation, using an enzyme immunoassay kit (BioCheck BC-1119). Kit instructions were modified for DBS as follows: one 3.2 mm disc of DBS specimen was isolated with a manual punch and eluted overnight in assay buffer at 4°C. Eluent was then assayed without further dilution. Dilution correction was performed assuming 1.5 μl of serum equivalent in a 3.2 mm disc [70]. The intra-assay coefficient of variation (CV) was 5.7% and inter-assay CV was 16.3% across 37 plates.

Anemia was defined as Hb <12 g/dl for women or <13 g/dl for men [71]. Diabetes was defined as HbA_1c_ ≥6.5%. Our sampling approach did not allow us to distinguish type 1 from T2D; however, no cases of type 1 diabetes were reported by participants in response to questions about chronic conditions, and, given limited access to care for complex medical issues, it is unlikely that many cases of type 1 diabetes were included in the sample [72]. Hypertension included stage 1 (130–139 mmHg systolic and/or 80–89 mmHg diastolic) and stage 2 (≥140 mmHg systolic and/or ≥90 mmHg diastolic) hypertension according to the American College of Cardiology guidelines [73]. Inflammation was assessed as a continuous variable with a maximum value of 10 mg/l and an ordinal variable denoting low (CRP < 3 mg/l), medium (3 mg/l ≤ CRP < 10 mg/l), and high (CRP ≥ 10 mg/l) inflammation [74].

### Statistical analysis

We estimated logistic regression models of associations between betel quid use and anemia, T2D, and hypertension. Findings from the anemia and diabetes analyses have been published separately and are reproduced here for their relevance to possible evolutionary explanations [62, 63]. We estimated a generalized linear model and an ordinal logistic regression model of the association between betel quid use and inflammation. We used cross-validation to identify the best model for CRP as a continuous variable (Table S2) and the Brant test (Table S3) to assess whether the ordinal logistic regression models violated the proportional odds assumption.

All multivariate models included age, gender, and the interaction between betel quid use and gender (given gender differences in disease risk and patterns of betel quid use). We evaluated a random intercept term for all models to test for clustering at the household level via intraclass correlation coefficient (Table S4). Additional variables considered as potential confounders included individual characteristics likely to be upstream of both betel quid use and chronic disease or inflammation, including SES and education, or that we suspected would systematically co-vary with betel quid use that were upstream of chronic disease or inflammation (food security and food source, smoking, access to improved toilet facilities). Control variables for each disease model were selected by cautious consideration of known risk factors to adequately identify the effect of betel. We did not control for variables (e.g. body mass index (BMI), grip strength) that were potentially on the causal path between betel quid and the outcomes of interest.

We evaluated dose–response relationships by estimating models with betel quid categories based on reported frequency of use: no betel use, infrequent betel use (monthly or weekly, but not daily), low use (1–4 times per day), and high use (≥5 times per day). We used R (version 4.2.0) statistical software for all analyses.

### Research ethics approval

Data collection and laboratory analyses were approved by both The Pennsylvania State University Institutional Review Board and the icddr,b Ethical Review Committee. Participants were made aware that the study was voluntary, briefed on the goals of the study and procedures, and provided written informed consent to participate. HbA_1c_ results (diabetic/not), Hb results (anemic/not), height, weight, BMI, and blood pressures (with indication of hypertension cutoffs) were reported to participants in writing at the time of data collection. All relevant data used for analyses are available upon request.

## RESULTS

Data for all four outcomes of interest (anemia, diabetes, hypertension, and inflammation) were available for 1115 people, 709 non-pregnant women, and 406 men (Table 1). The sample size differs slightly from our prior publications, as only those with complete data for all health outcomes were considered [62, 63]. Anemia was identified in 177 (43.6%) men and 345 (48.7%) women; diabetes was identified in 81 (20.0%) men and 94 (13.3%) women; hypertension was identified in 180 (44.3%) men and 436 (61.5%) women. High CRP was uncommon (16 men, 3.9%; 24 women, 3.4%). Betel quid use (any/none) was similar between men (53.2%) and women (53.6%). However, more men (33.3%) than women (20.9%) reported the highest frequency of betel use. A very small number of men and women (41) reported infrequent betel quid use; these were grouped with the “no use” category in models of betel quid use frequency.

**Table 1:** Characteristics of the study population.

	**Total *N* = 1115**	**Men *N* = 406**	**Women *N* = 709**	***N* missing**
**Categorical**	***N* (%)**			
Betel nut use No Betel nut use	596 (53.5) 519 (46.5)	216 (53.2) 190 (46.8)	380 (53.6) 329 (46.4)	0
Betel nut use ≥5 times per day Betel nut use <5 times per day Betel nut use less than daily No Betel nut use	283 (25.4) 259 (23.2) 43 (3.9) 519 (46.5)	132 (33.3) 73 (18.0) 8 (1.9) 190 (46.8)	148 (20.9) 186 (26.2) 35 (4.9) 329 (46.4)	11
Food secure Not food secure	751 (67.4) 364 (32.6)	280 (69.0) 126 (31.0)	471 (66.4) 238 (33.6)	0
All food from the bazaar Not all food from bazaar	637 (57.1) 477 (42.8)	192 (47.3) 213 (52.5)	445 (62.8) 264 (37.2)	1
Education No schooling Primary education Beyond primary education	358 (32.1) 379 (34.0) 377 (33.8)	114 (28.1) 136 (33.5) 155 (38.2)	244 (21.9) 243 (21.8) 222 (19.9)	1
Men who are laborers Men of other occupation	179 (44.1) 222 (54.7)	179 (44.1) 222 (54.7)		5
Tobacco use No tobacco use	162 (39.9) 244 (60.1)	162 (39.9) 244 (60.1)		0
Exposure to tobacco smoke No exposure to tobacco smoke	255 (36.0) 454 (64.0)		255 (36.0) 454 (64.0)	0
Toilet owned Toilet shared	238 (21.3) 873 (78.3)	87 (21.4) 317 (78.1)	151 (21.3) 556 (78.4)	4
Anemia No anemia	522 (46.8) 593 (53.2)	177 (43.6) 229 (56.4)	345 (48.7) 364 (51.3)	0
T2D No T2D	175 (15.7) 940 (84.3)	81 (20.0) 325 (80.0)	94 (13.3) 615 (86.7)	0
Hypertension No hypertension	616 (55.2) 499 (44.8)	180 (44.3) 226 (55.7)	436 (61.5) 273 (38.5)	0
CRP ≤ 3 (low) 3 < CRP < 10 (medium) CRP ≥ 10 (high)	834 (74.8) 241 (21.6) 40 (3.6)	313 (77.1) 77 (19.0) 16 (3.9)	521 (73.5) 164 (23.1) 24 (3.4)	0
**Continuous**	**Mean (SD)**			
Age	51.8 (12.3)	55.5 (11.8)	49.7 (12.1)	0
MacArthur Ladder Present	4.3 (1.7)	3.9 (1.2)	4.6 (1.8)	0
CRP (mg/l)	2.02 (1.84)	1.84 (1.73)	2.13 (1.90)	0

Men in the sample were older and had more years of education than women, while women had higher SES (by the MacArthur Scale of Subjective Social Status), and were more likely to obtain their food from the bazaar. These gender differences, as well as the difference in sample size, result in large part from labor migration by many men to Dhaka or abroad (places like Dubai, Saudi Arabia, Singapore, and Malaysia). Labor migrant husbands were younger, healthier, and wealthier than those who were present in Matlab at the time of data collection [1]. Thus, our sample captures the characteristics of men and women who currently reside in the study area, rather than those who could consider it their permanent or familial home.

### Anemia

**Figure 2:**
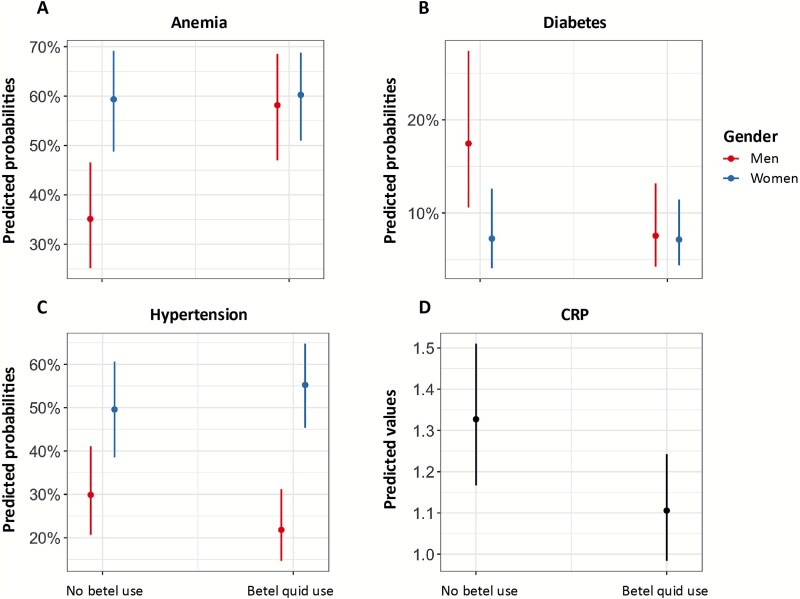
Predicted probabilities for anemia, diabetes, and hypertension, and predicted values for CRP.

Our analysis revealed a positive association between betel quid use and anemia (β = 0.94, *P*-value = 4.80e^−5^), with a significant interaction between betel quid use and gender (*P*-value = .001) such that the association is notable among men (aOR: 2.56; CI: 1.62, 4.04) but not women (aOR: 1.03; CI: 0.72, 1.49) (Table 2, Fig. 2A); this was true when betel quid use was considered as a binary or a categorical variable (for frequency of use; Supplemental information Tables S5 and S6). The association between frequency of betel quid use and anemia was robust to the categorization of “infrequent” use with either “no-use” or “low” use. Evaluation of a dose response found a positive association between frequency of betel quid use and anemia (β = 0.47, *P*-value = .0001) with a significant interaction between frequency of betel quid use and gender (*P*-value = .029) such that the positive association between increasing frequency of betel quid use and anemia was evident among men (aOR:1.61; CI:1.25, 2.06) and not women (aOR:1.13; CI: 0.91, 1.41) (Supplemental information Tables S6 and S7).

**Table 2:** Logistic regression models evaluating the association between betel quid use and anemia, diabetes, and hypertension, and generalized linear model evaluating the relationship between betel quid use and continuous CRP for men and women. The beta coefficients for the logistic regression models represent log odds.

		**Anemia**				**Diabetes**				**Hypertension**				**CRP continuous**			
		**β**	**P**	**OR**	**CI**	**β**	**P**	**OR**	**CI**	**β**	**P**	**OR**	**CI**	**β**	**P**	**OR**	**CI**
**Bivariable** **analyses**	Betel nut use	0.72	4.59e^−8^	2.04	1.58, 2.64	−0.05	0.79	0.95	0.68, 1.33	0.51	7.48e^−5^	1.66	1.29, 2.13	−0.22	0.051	0.80	0.64, 0.99
**Multivariable analyses**	Betel nut use	0.94	4.80e^−5^	2.56	1.62, 4.04	−0.95	0.001	0.38	0.22, 0.66	−0.42	0.070	0.65	0.41,1.03	−0.34	0.007	0.71	0.54, 0.92
	Women	0.99	6.94e^−6^	2.69	1.74, 4.15	−0.99	0.0002	0.36	0.22, 0.63	0.83	0.0001	2.31	1.50, 3.54	0.54	0.001	1.70	1.23, 2.37
	Betel nut use^*^gender	−0.91	0.001	0.40	0.23, 0.70	0.93	0.008	2.54	1.27, 5.10	0.65	0.022	1.92	1.09, 3.36				

### Diabetes

Betel quid use was inversely associated with diabetes (β = −0.95, *P*-value = .001), and there was a significant interaction effect between betel quid use and gender (*P*-value = .008) such that the association is evident among men (aOR: 0.38; CI: 0.22, 0.66) and not women (aOR: 0.98; CI: 0.58, 1.65) (Table 2, Fig. 2B). When frequency of betel quid use was categorized as a categorical variable, low and high daily betel quid use were associated with decreased odds of diabetes among men but not women (Supplemental information Table S6). This pattern was robust to the categorization of “infrequent” use with “no-use” or “low” use. Evaluation of dose response found an inverse association between frequency of betel quid use and diabetes (β = −0.55, *P*-value = .001) with a significant interaction between frequency of betel quid use and gender (*P*-value = .004) such that the inverse association between increasing betel quid exposure level and diabetes was evident among men (aOR: 0.57; CI: 0.42, 0.79) and not women (aOR: 1.05; CI: 0.77, 1.43) (Supplemental information Table S7).

### Hypertension

Betel quid use was inversely associated with hypertension (β = −0.42, *P*-value = .07), and there was a significant interaction between betel quid use and gender (*P*-value = .022) such that the inverse association is evident among men (aOR: 0.65; CI: 0.41, 1.03) but not women (aOR: 1.25; CI: 0.85, 1.85) (Table 2, Fig. 2C). When considering frequency of betel quid use as a categorical variable, high daily betel quid use was inversely associated with hypertension (Supplemental information Table S6). Evaluation of dose response found an inverse association between frequency of betel quid use and hypertension (β = −0.32, *P*-value = .016) with a significant interaction between frequency of betel quid use and gender (*P*-value = .005) such that the inverse association between increasing betel quid exposure level and hypertension is evident among men (aOR: 0.72; CI: 0.56, 0.94) and not women (aOR: 1.16; CI: 0.91, 1.48) (Supplemental information Table S7).

### Inflammation

Betel quid use was inversely associated with inflammation (β = −0.34; *P*-value = .007) and did not interact with gender (Table 2; Fig. 2D). When betel quid was categorized by frequency of use, low and high daily betel quid use were inversely associated with CRP compared with no betel quid use (β = −0.18; *P*-value = .028, β = −0.27; *P*-value = .001, respectively; Supplemental information Table S9). Evaluation of dose response found an inverse association between frequency of betel quid use and CRP (β = −0.13, *P*-value = .001) (Supplemental information Table S10; Tables S19–S23). Ordinal logistic regression models of CRP showed similar patterns: betel quid use as a binary or categorical variable was inversely associated with inflammation (Supplemental Tables S8–S10).

For all the outcomes, models stratified by gender were generally similar to those that assessed interaction terms (Supplement Information Tables S11–S18).

## DISCUSSION

The ubiquity of psychoactive substances globally raises questions about the motivations and contexts behind their use. Research has focused primarily on investigating the neurological pathways underlying addiction and the systemic health consequences of chronic substance use [11, 75]. Though warranted, this focus may overlook the motivations for traditional use of psychoactive substances prior to the modern refinement that has, for many substances, exacerbated addiction and its associated consequences. We examined the relationship between betel quid use and four health outcomes among a representative cross-sectional sample of women and their husbands in rural Bangladesh. Our findings do not support the hypothesis that betel quid use would result in adverse effects for diseases that have become more prevalent relatively recently. Instead, we find protective associations with diabetes, hypertension, and inflammation (a risk factor of cardiometabolic disorders), suggesting that betel quid’s health effects are more complex than previously reported and prompting consideration of potential evolutionary reasons for its use, and importantly, its plausible interaction with mechanisms underlying these health outcomes.

### Public health implications of betel quid use

Prior work from our group highlights the complex relationship between betel quid and health. In the context of rural Bangladesh, we found stronger adverse effects of betel quid use on anemia among men than (non-pregnant) women [62], aligning with studies showing increased risk for anemia among pregnant women with betel quid use [76, 77]. Though mechanisms causing anemia remain unclear, it has been hypothesized that mechanical damage to oral mucosa from chewing betel quid or irritation of the gastrointestinal tract may result in blood loss [78, 79]. Betel quid use may also contribute to anemia via iron deficiency, though this was not evident in our analyses [62]. Finally, field observations suggest that chewing betel quid suppresses appetite and affects the taste of food, thus reducing the enjoyment and intake of food, contributing to nutrient deficiencies and anemia [80].

In the same dataset, we found an inverse association between betel quid use and diabetes that was stronger among men, which persisted after consideration of confounding [63]. We considered multiple mediation routes (e.g. through lower BMI), but identified no pathways, supporting a direct effect of betel quid ingredients on diabetes risk. Our findings contrast with studies in Taiwan [58, 60] and among Bangladeshi women in London [81], which reported positive associations between betel quid and diabetes. It is difficult to consider these findings discrepant, however, as the very different ecologies in which betel quid is used in Taiwan, London, and rural Bangladesh may contribute to its differential effects. Specifically, different betel quid components and local preparations, such as the more common use of ripe, cured betel nut throughout South Asia versus fresh, unripe nut used in Taiwan, may interact differently with local diets and exposures to modulate health outcomes [82].

Animal models provide some nuance on betel quid’s effects, suggesting that some betel quid components may lower diabetes risk (e.g. suppressing post-prandial glucose elevation) [83]. Based on these studies and our findings from Matlab, we posit that betel quid may protect against diabetes among our participants via subtle effects on energy use or absorption. To date, animal, *in vitro*, and in silico studies propose three mechanisms for betel quid’s hypoglycemic effects: (1) altering intestinal sugar absorption, (2) promoting insulin secretion or mimicking insulin activity, and 3) modulating enzymes involved in gluconeogenesis and glycogenesis (Table 3) [83–86].

**Table 3:** The literature shows positive associations between betel quid components and regulation of blood glucose levels by way of several mechanisms.

**Major components of betel quid**	**Mechanisms implicated in regulation of blood glucose levels**			
	**Decreased intestinal sugar absorption**	**Regulating insulin and/or glucagon secretion**	**Regulating gluconeogenesis and/or glycogenesis**	**Unspecified**
Betel nut (*A. catechu*)	Betel nut extract inhibits intestinal absorption of monosaccharides, resulting in reduced postprandial blood glucose levels [78].	Arecoline may prevent pancreatic beta cell dysfunction by up-regulating pancreatic and duodenal homeobox 1 (PDX-1), a key regulator of insulin secretion [95].	Betel nut procyanidins regulate gluconeogenesis by reducing expression of two key enzymes (phosphoenol-pyruvate carboxykinase and glucose-6-phosphatase) [79].	Betel nut contains alkaloids (arecoline), which may have antidiabetic effects, including lowering blood glucose levels [59].
Betel leaf (*P. betle*)	Reduced blood glucose via inhibition of alpha-amylase and alpha-glucosidase, two enzymes that support the intestinal breakdown and absorption of glucose [80, 96, 97].	Increased storage of glycogen in liver and muscles through insulinomimetic effects [98].	Betel leaf extracts decrease liver fructose-1,6-bisphosphatase and glucose-6-phosphatase activity, which support gluconeogenesis. Extracts were also found to increase hexokinase activity promoting glycolysis or glycogen synthesis [81].	Anti-diabetic effect by decreasing glucose absorption in the gut, regulating insulin, or driving glucose uptake in the tissue [99].

Our analysis of hypertension and inflammation (as measured by CRP) further highlights the importance of considering betel quid use’s impact on health in population-specific context. We found an inverse association between betel quid use and hypertension among men, contrasting with findings from Araihazar, Bangladesh, where researchers found a positive association between betel quid use and hypertension, particularly among women [67]. Differences in hypertension cut-offs between the Araihazar study and this study may partly explain this discrepancy. Conversely, a study of hypertension in Pakistan found no association with betel nut without additional ingredients but did find a positive association when tobacco was added to the quid [87]. Until research provides a more thorough understanding of variation in betel quid content and how betel use is influenced by socioecological context, it remains unclear whether these contrasting findings reflect true inconsistencies or result from heterogeneity in betel quid composition or other factors.

Betel quid’s effects on blood pressure may be driven by both physiological and psychosocial mechanisms [88]. First, areca nut metabolites have been found to inhibit angiotensin converting enzyme (ACE), a key regulator of electrolyte balance and blood pressure [89]. The inhibitory properties of these metabolites on ACE were comparable to captopril, one of the early ACE inhibitors. Second, betel quid is often used to reduce stress, with a study from Taiwan identifying “having greater perceived stress” as a common reason for use [90, 91]. Chronic stress conditions (from SES, occupation/work, discrimination, etc.) may drive continuous activation of the sympathetic nervous system or hormonal cascades such as the renin-angiotensin-aldosterone system (RAAS, a pathway that uses ACE to regulate blood pressure), resulting in persistent elevated blood pressure and endothelial damage [92]. Finally, the preparation and chewing of betel quid is often a social act that may relieve stress, particularly to the extent that it is consumed with peers and so provides an opportunity for social bonding.

The sex differences observed in our study may reflect known sex differences in the development of hypertension. Specifically, estrogen and testosterone modulate the RAAS pathway to affect blood pressure regulation in women and men, respectively [93]. Thus, betel quid’s different effects on hypertension in women and men may reflect interactions with this hormone-sensitive system. Additionally, cultural factors that affect patterns of betel quid use among women and men may also contribute to sex differences. The use of betel quid is more frequent among men than women, and thus the social dynamics around its use may differ between the two, affecting the benefits gleaned from the ritual use of betel quid.

Finally, the differences across studies and populations may also be driven by gene-by-environment interactions, i.e. genetic susceptibility may modify disease risk among chewers [94]. The gene encoding ACE has a well-known polymorphism represented by an insertion (I allele) or deletion (D allele), with increased risk for hypertension conferred by the D allele [95]. Chung et al. found that betel quid chewers with the D allele had significantly lower systolic blood pressure and reduced hypertension risk compared to non-chewers with the same allele [96].

In our sample, betel quid was inversely associated with inflammation. While most studies report positive associations between betel quid and inflammation, including one in Pakistan linking betel quid and tobacco additives to higher CRP in older men [87], animal and *in vitro* studies have shown anti-inflammatory effects of betel nut metabolites [84, 97, 98]. These findings may be reconcilable, however, as the non-human studies have identified anti-inflammatory compounds in betel nut, but there is also inflammation caused by “real life” use of betel quid (e.g. oral damage caused by chewing hard or abrasive components of the quid), potentially resulting in a net increase in inflammation in most human studies to date. These findings highlight the complexity of betel quid, whose therapeutic and harmful effects may vary by preparation, physiology, and usage patterns.

### Betel quid use in an evolutionary context

Our evidence is consistent with both fitness costs (anemia) and benefits (lower inflammation, protection against diabetes and hypertension) to betel quid use among adults living in rural Bangladesh. These contrasting effects suggest that trade-offs may have been a driving factor in the use of betel quid in the past. Our findings prompt several evolutionary explanations for the widespread adoption of betel quid use in this area.

Immune responses, especially inflammation, are “high-cost, high-benefit” traits [99] that are energetically expensive and potentially damaging, yet essential to survival. When white blood cells recognize pathogens, they activate nonspecific responses that potentiate further immune defense against infection [100] and can damage host tissues (e.g. the production of reactive oxygen species by macrophages) [101, 102]. In Bangladesh, an environment with a heavy infectious disease burden, costs of inflammation may be amplified. Betel quid use may have benefitted survival and/or lengthened lifespan prior to disability if the anti-inflammatory metabolites [103–106] limited collateral damage to tissues during frequent immune activation.

Within the pathogen-rich setting of Bangladesh (and more broadly since our transition to consistent meat-eating behavior), helminthic infections have been common and may have contributed to the adoption and spread of betel quid use. Arecoline, betel quid’s main psychoactive metabolite, has demonstrated anthelmintic effects in both humans and non-human animals [107, 108]. Lower inflammation among betel quid chewers may reflect reduced helminth burden. Given the strong evolutionary pressures posed by pathogens across human evolutionary history, betel quid use may have been an adaptive means for individuals to mitigate these infections and their consequences.

While diabetes itself is a recent phenomenon, betel quid’s metabolic effects may offer evolutionarily relevant benefits. Betel quid use may delay depletion of energy stores by modulating key enzymes involved in gluconeogenesis and glycogenesis, resulting in a reduction of circulating blood glucose (Table 3) [78, 80]. Specifically, betel quid components have been found to downregulate phosphoenolpyruvate carboxykinase and glucose-6-phosphatase, enzymes crucial to the production of glucose in the liver. Moreover, arecoline promotes glucose storage via insulin-like properties. Together, these actions may reduce overall glucose availability, restrict the availability of energy, or promote energy storage during sudden, large, prolonged increases in expenditure. This may be particularly relevant in supporting immune defense, which is metabolically expensive and can deplete energy stores [92]. In high-infection settings, betel quid may have helped buffer the energetic costs of immunity.

While we find a positive association between betel quid use and anemia, and an inverse association between betel quid use and diabetes, these findings may be confounded by unmeasured variables such as helminthic infections. That is, helminthic infections could be contributing to anemia in our population, and the observed relationship between betel quid and anemia may simply reflect self-medication behavior among anemic participants. Helminthic infections have also been associated with improved metabolic function (e.g. decreased fasting blood glucose and glycated Hb) [109, 110]. Thus, the inverse relationship between betel quid use and diabetes may also reflect confounding by helminth infections. Without direct measures of infections or other unmeasured variables, we cannot rule out reverse causation or confounding.

An evolutionary explanation for betel quid’s effects on hypertension centers around the observation that a gene-by-environment interaction may underlie betel quid’s anti-hypertensive properties. Individuals with the D haplotype in the ACE gene produce more ACE, leading to increased vascular tone and overall electrolyte retention, resulting in elevated blood pressure and increased risk of hypertension [95, 111, 112]. The capacity for increased salt and water retention may have been a beneficial trait that maintained the frequency of this allele in early human populations, with the by-product consequence of hypertension in modern contexts [113]. Thus, in changing environments in which this trait was not favorable, consumption of betel quid (whose extracts have been found to inhibit ACE) may offset its deleterious effects. Our observation that betel quid use was positively associated with anemia highlights the importance of considering evolutionary tradeoffs. That is, the optimization of one trait or behavior may come at the expense of another as organisms and systems face physiological and resource constraints [114]. In rural Bangladesh, consumption of betel quid may be driven by its ability to reduce inflammation, promote energy storage, and/or promote electrolyte balance at the expense of byproduct consequences for other physiological systems, including risk for anemia.

### Limitations

The observational and cross-sectional nature of our data limits our ability to make causal inferences. Our survey data, though thoroughly pre-tested and carefully collected, run the risk of recall bias. Additionally, we did not collect information about participants’ typical betel quid preparation, preventing us from attributing the observed effects strictly to betel nut or any specific component. Betel quid’s preparation varies greatly across and within populations where its use is common, and different combinations include variation in areca nut ripeness, betel leaf, and other ingredients [82]. This variation in preparation, along with potential interactions between different betel quid components and local diets, may explain some heterogeneity across studies. For the CRP analysis, we were unable to distinguish between acute and chronic elevations in inflammation. We also acknowledge the possibility of reverse causation or confounding that may be driven by unmeasured variables such as helminthic infections, which have been independently associated with both the use of betel quid and our health outcomes. Finally, the generalizability of our findings beyond rural Bangladesh remains to be evaluated.

### Conclusions

Betel quid use has complex effects on health in rural Bangladesh, increasing risk for some conditions (anemia) and seemingly decreasing risk for others (diabetes and hypertension among men, and inflammation broadly). Overall, these patterns implicate evolutionary trade-offs in the use of betel quid. In historical contexts, betel quid use may have reduced the inflammatory damage and energetic costs of immune defense, reduced inflammation by treating helminthic infections, promoted efficient use of nutrients during times of resource uncertainty, and/or supported electrolyte balance. Application of an evolutionary framework to the complex relationship between betel quid use and health offers a valuable model to investigate how the traditional use of other psychoactive substances may represent behavioral adaptations to diverse socioecological challenges.

## Supplementary Material

eoaf037_EMPH_Supplementary_Tables_Final
