# Supplementary material for: An evolutionary perspective on the use of betel nut and its effects on health outcomes
Source: Evol Med Public Health. 2025 Dec 15;14(1):1–15. doi: 10.1093/emph/eoaf037 (PMC12887317; doi:10.1093/emph/eoaf037)
Supplement: eoaf037_EMPH_Supplementary_Tables_Final [file eoaf037_emph_supplementary_tables_final.docx]

**SUPPLEMENTAL TABLES**

**Table S1.** Reasons for attrition after the 2010 study.

| **Reasons for attrition** | **Count** |
| --- | --- |
| Moving out of the area | 154 |
| Death | 4 |
| Disability | 14 |
| Decline | 4 |
| Travel outside of Bangladesh | 3 |

**Table S2.** Evaluating best “family” (i.e., distribution and link function) for CRP as a continuous variable through cross validation (CV).

| **Model** | **CV error** |
| --- | --- |
| CRP, gaussian | 3.37 |
| CRP, gamma | 3.36 |
| CRP, gaussian log link | 3.39 |
| CRP, gamma log link | 3.37 |

**Table S3.** Brant test for violation of the proportional odds assumption in ordinal logistic regression models of CRP.

| **Variables** | **P-value** | | |
| --- | --- | --- | --- |
|  | **Both** | **Women** | **Men** |
| Omnibus | 0.78 | 0.69 | 0.46 |
| Betel nut use | 0.66 | 0.67 | 0.39 |
| Female | 0.29 |  |  |
| Age | 0.90 | 0.73 | 0.74 |
| MacArthur’s Ladder Present | 0.92 | 0.97 | 0.59 |
| Tobacco use (exposure to tobacco smoke) | 0.16 | 0.21 | 0.56 |
| Food from the bazar | 0.43 | 0.71 | 0.48 |
| Toilet owned or shared | 0.99 | 0.21 | 0.11 |

**Table S4.** Intraclass correlation coefficient (ICC) to evaluate clustering effect with models that include both men and women.

| **Outcome** | **ICC** |
| --- | --- |
| Anemia | 0.058* |
| Diabetes | 0.106* |
| Hypertension | 0.082* |
| CRP continuous | 0.051* |
| CRP binary | 5.32e^-13^ |
| CRP ordinal | 2.53e^-9^ |
| **Significant ICC (above 0.5) indicating possible clustering effect on the outcome variable.* | |

**Table S5.** Full logistic regression models evaluating the association between betel quid use (binary yes/no) and anemia, diabetes, and hypertension for men and women. The beta coefficients for the logistic regression models represent log odds.

|  | | **Anemia** | | | | **Diabetes** | | | | **Hypertension** | | | |
| --- | --- | --- | --- | --- | --- | --- | --- | --- | --- | --- | --- | --- | --- |
|  |  | **β** | **P** | **OR** | **CI** | **β** | **P** | **OR** | **CI** | **β** | **P** | **OR** | **CI** |
| **Bivariable analyses** | Betel nut use | 0.72 | 4.59e^-8^ | 2.04 | 1.58, 2.64 | -0.05 | 0.79 | 0.95 | 0.68, 1.33 | 0.51 | 7.48e^-5^ | 1.66 | 1.29, 2.13 |
|  | | | | | | | | | | | | | |
| **Multivariable**  **analyses** | Betel nut use | 0.94 | 4.80e^-5^ | 2.56 | 1.62, 4.04 | -0.95 | 0.001 | 0.38 | 0.22, 0.66 | -0.42 | 0.070 | 0.65 | 0.41,1.03 |
|  | Women | 0.99 | 6.94e^-6^ | 2.69 | 1.74, 4.15 | -0.99 | 0.0002 | 0.36 | 0.22, 0.63 | 0.83 | 0.0001 | 2.31 | 1.50, 3.54 |
|  | Betel nut use*Women | -0.91 | 0.001 | 0.40 | 0.23, 0.70 | 0.93 | 0.008 | 2.54 | 1.27, 5.10 | 0.65 | 0.022 | 1.92 | 1.09, 3.36 |
|  | Age | 0.03 | 2.42e^-6^ | 1.03 | 1.02, 1.05 | 0.03 | 5.64e^-5^ | 1.04 | 1.02, 1.05 | 0.05 | 2.80e^-13^ | 1.06 | 1.04, 1.07 |
|  | MacArthur Ladder Present | -0.10 | 0.022 | 0.91 | 0.83, 0.98 | 0.15 | 0.006 | 1.16 | 1.05, 1.30 | 0.00 | 0.992 | 1.00 | 0.92, 1.09 |
|  | Primary education | -0.35 | 0.031 | 0.70 | 0.51, 0.96 | -0.13 | 0.559 | 0.88 | 0.57, 1.34 | 0.25 | 0.159 | 1.28 | 0.91, 1.82 |
|  | Beyond primary education | -0.08 | 0.639 | 0.91 | 0.63, 1.32 | -0.26 | 0.275 | 0.77 | 0.47, 1.23 | 0.32 | 0.100 | 1.38 | 0.93, 2.05 |
|  | Exposure to tobacco smoke | -0.44 | 0.001 | 0.64 | 0.49, 0.84 |  |  |  |  | 0.24 | 0.095 | 1.27 | 0.96, 1.69 |
|  | Food secure | 0.07 | 0.604 | 1.08 | 0.81, 1.44 | 0.59 | 0.005 | 1.80 | 1.19, 2.74 | 0.32 | 0.049 | 1.37 | 1.00, 1.87 |
|  | Bazar food | -0.17 | 0.203 | 0.84 | 0.64, 1.09 | 0.43 | 0.017 | 1.55 | 1.08, 2.22 | 0.13 | 0.373 | 1.13 | 0.85, 1.51 |

|  | | **Anemia** | | | | **Diabetes** | | | | **Hypertension** | | | |
| --- | --- | --- | --- | --- | --- | --- | --- | --- | --- | --- | --- | --- | --- |
|  |  | **β** | **P** | **OR** | **CI** | **β** | **P** | **OR** | **CI** | **β** | **P** | **OR** | **CI** |
| **Bivariable analyses** | Daily low | 0.78 | 1.57e^-6^ | 2.18 | 1.58, 3.00 | -0.10 | 0.622 | 0.90 | 0.59, 1.36 | 0.61 | 0.000 | 1.84 | 1.34, 2.54 |
|  | Daily high | 0.82 | 2.40e^-7^ | 2.27 | 1.66, 3.10 | -0.06 | 0.757 | 0.93 | 0.63, 1.39 | 0.28 | 0.063 | 1.33 | 0.98, 1.80 |
|  | | | | | | | | | | | | | |
| **Multivariable**  **analyses** | Daily low | 1.41 | 9.66e^-6^ | 4.12 | 2.20, 7.71 | -1.43 | 0.001 | 0.23 | 0.10, 0.55 | -0.22 | 0.487 | 0.80 | 0.43, 1.49 |
|  | Daily high | 0.93 | 0.000 | 2.52 | 1.52, 4.16 | -1.00 | 0.001 | 0.36 | 0.19, 0.67 | -0.64 | 0.015 | 0.52 | 0.31, 0.88 |
|  | Women | 0.99 | 3.45e^-6^ | 2.71 | 1.77, 4.11 | -1.11 | 1.86e^-5^ | 0.32 | 0.19, 0.54 | 0.84 | 6.76e^-5^ | 2.33 | 1.53, 3.55 |
|  | Daily low* Women | -1.35 | 0.000 | 0.25 | 0.12, 0.53 | 1.45 | 0.003 | 4.28 | 1.61, 11.3 | 0.32 | 0.383 | 1.38 | 0.66, 2.88 |
|  | Daily high* Women | -0.61 | 0.059 | 0.54 | 0.28, 1.02 | 1.06 | 0.011 | 2.91 | 1.27, 6.62 | 0.98 | 0.004 | 2.66 | 1.34, 5.28 |
|  | Age | 0.03 | 7.77e^-6^ | 1.03 | 1.02, 1.05 | 0.04 | 6.07e^-5^ | 1.03 | 1.02, 1.05 | 0.05 | 1.38e^-13^ | 1.06 | 1.04, 1.07 |
|  | MacArthur Ladder Present | -0.09 | 0.024 | 0.91 | 0.83, 0.99 | 0.16 | 0.005 | 1.17 | 1.04, 1.31 | 0.01 | 0.918 | 1.00 | 0.91, 1.10 |
|  | Primary education | -0.38 | 0.020 | 0.68 | 0.49, 0.94 | -0.18 | 0.411 | 0.83 | 0.54, 1.28 | 0.24 | 0.183 | 1.27 | 0.89, 1.82 |
|  | Beyond primary education | -0.06 | 0.719 | 0.93 | 0.64, 1.35 | -0.29 | 0.234 | 0.74 | 0.46, 1.21 | 0.30 | 0.135 | 1.35 | 0.91, 2.01 |
|  | Exposure to tobacco smoke | -0.47 | 0.001 | 0.62 | 0.47, 0.81 |  |  |  |  | 0.21 | 0.150 | 1.23 | 0.93, 1.65 |
|  | Food secure | 0.08 | 0.557 | 1.09 | 0.81, 1.47 | 0.55 | 0.009 | 1.74 | 1.14, 2.65 | 0.33 | 0.038 | 1.40 | 1.01, 1.92 |
|  | Bazar food | -0.15 | 0.249 | 0.85 | 0.65, 1.11 | 0.39 | 0.032 | 1.48 | 1.27, 2.13 | 0.13 | 0.345 | 1.14 | 0.86, 1.53 |

**Table S6.** Full logistic regression models evaluating the association between frequency of betel quid use (categorical) and anemia, diabetes, and hypertension for men and women. The beta coefficients for the logistic regression models represent log odds.

**Table S7.** Full logistic regression models evaluating the association between frequency of betel quid use (ordinal) and anemia, diabetes, and hypertension for men and women. The beta coefficients for the logistic regression models represent log odds.

|  | | **Anemia** | | | | **Diabetes** | | | | **Hypertension** | | | |
| --- | --- | --- | --- | --- | --- | --- | --- | --- | --- | --- | --- | --- | --- |
|  |  | **β** | **P** | **OR** | **CI** | **β** | **P** | **OR** | **CI** | **β** | **P** | **OR** | **CI** |
| **Bivariable analyses** | Betel nut use | 0.44 | 2.24e^-8^ | 1.55 | 1.33, 1.81 | -0.03 | 0.707 | 0.96 | 0.79, 1.17 | 0.18 | 0.017 | 1.20 | 1.03, 1.40 |
|  | | | | | | | | | | | | | |
| **Multivariable**  **analyses** | Betel nut use | 0.47 | 0.0001 | 1.61 | 1.25, 2.06 | -0.55 | 0.001 | 0.57 | 0.42, 0.79 | -0.32 | 0.016 | 0.72 | 0.56, 0.94 |
|  | Women | 0.83 | 3.56e^-5^ | 2.29 | 1.54, 3.41 | -1.03 | 3.95e^-5^ | 0.35 | 0.21, 0.58 | 0.83 | 5.47e^-5^ | 2.29 | 1.53, 3.43 |
|  | Betel nut use* Women | -0.35 | 0.029 | 0.70 | 0.51, 0.96 | 0.60 | 0.004 | 1.82 | 1.20, 2.77 | 0.47 | 0.005 | 1.61 | 1.14, 2.24 |
|  | Age | 0.03 | 1.66e^-6^ | 1.03 | 1.02, 1.04 | 0.03 | 0.0001 | 1.03 | 1.02, 1.05 | 0.05 | 4.14e^-14^ | 1.06 | 1.04, 1.08 |
|  | MacArthur Ladder Present | -0.10 | 0.017 | 0.90 | 0.83, 0.98 | 0.15 | 0.005 | 1.17 | 1.03, 1.31 | 0.003 | 0.943 | 1.00 | 0.91, 1.09 |
|  | Primary education | -0.38 | 0.021 | 0.68 | 0.49, 0.94 | -0.17 | 0.43 | 0.84 | 0.55, 1.29 | 0.24 | 0.182 | 1.27 | 0.89, 1.81 |
|  | Beyond primary education | -0.08 | 0.656 | 0.92 | 0.64, 1.33 | -0.25 | 0.285 | 0.77 | 0.48, 1.24 | 0.30 | 0.132 | 1.35 | 0.91, 2.00 |
|  | Exposure to tobacco smoke | -0.42 | 0.002 | 0.66 | 0.50, 0.86 |  |  |  |  | 0.22 | 0.133 | 1.24 | 0.93, 1.66 |
|  | Food secure | 0.11 | 0.470 | 1.11 | 0.83, 1.49 | 0.54 | 0.010 | 1.72 | 1.12, 2.62 | 0.34 | 0.036 | 1.41 | 1.02, 1.94 |
|  | Bazar food | -0.16 | 0.22 | 0.84 | 0.51, 0.96 | 0.41 | 0.025 | 1.51 | 1.05, 2.16 | 0.13 | 0.355 | 1.14 | 0.86, 1.52 |

**Table S8.** Full generalized linear model, logistic regression model, and ordinal logistic regression model evaluating the association between betel quid use (binary yes/no) and CRP as a continuous, binary, and ordinal variable for men and women. The beta coefficients for the logistic regression models represent log odds.

|  | | **CRP continuous** | | | | **CRP binary** | | | | **CRP ordinal** | | | |
| --- | --- | --- | --- | --- | --- | --- | --- | --- | --- | --- | --- | --- | --- |
|  |  | **β** | **P** | **OR** | **CI** | **β** | **P** | **OR** | **CI** | **β** | **P** | **OR** | **CI** |
| **Bivariable analyses** | Betel nut use | -0.22 | 0.051 | 0.80 | 0.63, 0.99 | -0.29 | 0.041 | 0.74 | 0.55, 0.98 | -0.29 | 0.042 | 0.74 | 0.55, 0.99 |
|  | | | | | | | | | | | | | |
| **Multivariable**  **analyses** | Betel nut use | -0.34 | 0.007 | 0.71 | 0.54, 0.92 | -0.36 | 0.036 | 0.69 | 0.49, 0.97 | -0.36 | 0.037 | 0.69 | 0.49, 0.97 |
|  | Women | 0.54 | 0.001 | 1.70 | 1.22, 2.37 | 0.21 | 0.208 | 1.23 | 0.88, 1.73 | 0.22 | 0.206 | 1.23 | 0.88, 1.73 |
|  | Age | 0.01 | 0.051 | 1.01 | 1.00, 1.02 | 0.00 | 0.703 | 1.00 | 0.98, 1.02 | 0.002 | 0.704 | 1.00 | 0.98, 1.02 |
|  | MacArthur Ladder Present | 0.07 | 0.056 | 1.07 | 1.00, 1.15 | 0.10 | 0.026 | 1.10 | 1.01, 1.21 | 0.10 | 0.026 | 1.10 | 1.01, 1.21 |
|  | Exposure to tobacco smoke | 0.22 | 0.054 | 1.25 | 1.00, 1.58 | 0.51 | 0.001 | 1.66 | 1.23, 2.23 | 0.50 | 0.001 | 1.65 | 1.22, 2.23 |
|  | Bazar food | 0.17 | 0.130 | 1.18 | 0.95, 1.48 | 0.24 | 0.118 | 1.27 | 0.94, 1.72 | 0.24 | 0.163 | 1.27 | 0.94, 1.72 |
|  | Toilet owned or shared | -0.24 | 0.066 | 0.78 | 0.61, 1.03 | -0.12 | 0.536 | 0.88 | 0.61, 1.29 | -0.12 | 0.533 | 0.88 | 0.61, 1.29 |

**Table S9.** Full generalized linear model, logistic regression model, and ordinal logistic regression model evaluating the association between frequency of betel quid use (categorical) and CRP as a continuous, binary, and ordinal variable for men and women. The beta coefficients for the logistic regression models represent log odds.

|  | | **CRP continuous** | | | | **CRP binary** | | | | **CRP ordinal** | | | |
| --- | --- | --- | --- | --- | --- | --- | --- | --- | --- | --- | --- | --- | --- |
|  |  | **β** | **P** | **OR** | **CI** | **β** | **P** | **OR** | **CI** | **β** | **P** | **OR** | **CI** |
| **Bivariable analyses** | Daily low | -0.05 | 0.431 | 0.94 | 0.81, 1.09 | -0.31 | 0.094 | 0.73 | 0.51, 1.05 | -0.31 | 0.094 | 0.73 | 0.51, 1.05 |
|  | Daily high | -0.22 | 0.002 | 0.79 | 0.69, 0.92 | -0.41 | 0.027 | 0.66 | 0.46, 0.95 | -0.40 | 0.028 | 0.66 | 0.46, 0.95 |
|  | | | | | | | | | | | | | |
| **Multivariable**  **analyses** | Daily low | -0.18 | 0.028 | 0.83 | 0.71, 0.98 | -0.46 | 0.027 | 0.62 | 0.41, 0.95 | -0.46 | 0.027 | 0.62 | 0.42, 0.95 |
|  | Daily high | -0.27 | 0.001 | 0.76 | 0.65, 0.89 | -0.39 | 0.053 | 0.67 | 0.44,1.01 | -0.39 | 0.057 | 0.67 | 0.45, 1.01 |
|  | Women | 0.14 | 0.034 | 1.15 | 1.01, 1.31 | 0.20 | 0.238 | 1.22 | 0.87, 1.71 | 0.20 | 0.234 | 1.22 | 0.87, 1.71 |
|  | Age | 0.01 | 0.004 | 1.01 | 1.00, 1.01 | 0.00 | 0.580 | 1.00 | 0.98, 1.02 | 0.004 | 0.581 | 1.00 | 0.98, 1.02 |
|  | MacArthur Ladder Present | 0.02 | 0.128 | 1.02 | 0.99, 1.07 | 0.10 | 0.027 | 1.11 | 1.01, 1.21 | 0.10 | 0.027 | 1.11 | 1.01, 1.21 |
|  | Exposure to tobacco smoke | 0.08 | 0.178 | 1.08 | 0.96, 1.23 | 0.53 | 0.0004 | 1.71 | 1.26, 2.31 | 0.53 | 0.001 | 1.70 | 1.26, 2.29 |
|  | Bazar food | 0.13 | 0.069 | 1.12 | 0.99, 1.26 | 0.22 | 0.145 | 1.25 | 0.92, 1.70 | 0.23 | 0.142 | 1.25 | 0.93, 1.71 |
|  | Toilet owned or shared | -0.14 | 0.059 | 0.86 | 0.74, 1.01 | -0.08 | 0.646 | 0.91 | 0.62, 1.33 | -0.09 | 0.642 | 0.91 | 0.62, 1.33 |

**Table S10.** Full generalized linear model, logistic regression model, and ordinal logistic regression model evaluating the association between frequency of betel quid use (ordinal) and CRP as a continuous, binary, and ordinal variable for men and women. The beta coefficients for the logistic regression models represent log odds.

|  | | **CRP continuous** | | | | **CRP binary** | | | | **CRP ordinal** | | | |
| --- | --- | --- | --- | --- | --- | --- | --- | --- | --- | --- | --- | --- | --- |
|  |  | **β** | **P** | **OR** | **CI** | **β** | **P** | **OR** | **CI** | **β** | **P** | **OR** | **CI** |
| **Bivariable analyses** | Betel nut use | -0.11 | 0.003 | 0.89 | 0.83, 0.96 | -0.21 | 0.017 | 0.80 | 0.67, 0.96 | -0.21 | 0.018 | 0.81 | 0.67, 0.96 |
|  | | | | | | | | | | | | | |
| **Multivariable**  **analyses** | Betel nut use | -0.13 | 0.001 | 0.87 | 0.81, 0.94 | -0.21 | 0.041 | 0.81 | 0.65, 0.99 | -0.21 | 0.043 | 0.81 | 0.660.99 |
|  | Women | 0.13 | 0.042 | 1.14 | 1.01, 1.29 | 0.16 | 0.336 | 1.17 | 0.84, 1.63 | 0.16 | 0.333 | 1.17 | 0.84, 1.63 |
|  | Age | 0.01 | 0.005 | 1.01 | 1.00, 1.01 | 0.001 | 0.848 | 1.00 | 0.98, 1.02 | 0.001 | 0.851 | 1.00 | 0.98, 1.02 |
|  | MacArthur Ladder Present | 0.02 | 0.131 | 1.02 | 0.99, 1.06 | 0.09 | 0.030 | 1.11 | 1.01, 1.21 | 0.09 | 0.031 | 1.10 | 1.00, 1.21 |
|  | Exposure to tobacco smoke | 0.08 | 0.197 | 1.08 | 0.96, 1.22 | 0.51 | 0.001 | 1.67 | 1.24, 2.25 | 0.51 | 0.001 | 1.66 | 1.23, 2.24 |
|  | Bazar food | 0.11 | 0.068 | 1.12 | 0.99, 1.26 | 0.23 | 0.135 | 1.26 | 0.93, 1.71 | 0.23 | 0.133 | 1.26 | 0.93, 1.72 |
|  | Toilet owned or shared | -0.14 | 0.058 | 0.86 | 0.74, 1.01 | -0.09 | 0.633 | 0.91 | 0.62, 1.33 | -0.09 | 0.629 | 0.91 | 0.62, 1.33 |

| **Women** | | **Anemia** | | | | **Diabetes** | | | | **Hypertension** | | | |
| --- | --- | --- | --- | --- | --- | --- | --- | --- | --- | --- | --- | --- | --- |
|  |  | **β** | **P** | **OR** | **CI** | **β** | **P** | **OR** | **CI** | **β** | **P** | **OR** | **CI** |
| **Bivariable analyses** | Betel nut use | 0.45 | 0.002 | 1.58 | 1.17, 2.12 | 0.48 | 0.033 | 1.62 | 1.04, 2.57 | 0.81 | 3.11e^-7^ | 2.23 | 1.64, 3.05 |
|  | | | | | | | | | | | | | |
| **Multivariable**  **analyses** | Betel nut use | 0.22 | 0.247 | 1.24 | 0.85, 1.82 | -0.18 | 0.518 | 0.83 | 0.47, 1.46 | 0.05 | 0.770 | 1.06 | 0.71, 1.57 |
|  | Age | 0.02 | 0.011 | 1.02 | 1.01, 1.04 | 0.03 | 0.002 | 1.03 | 1.01, 1.06 | 0.06 | 1.34e^-9^ | 1.06 | 1.04, 1.08 |
|  | MacArthur Ladder Present | -0.09 | 0.029 | 0.91 | 0.82, 0.98 | 0.10 | 0.098 | 1.11 | 0.97, 1.26 | -0.02 | 0.590 | 0.97 | 0.88, 1.07 |
|  | Primary education | -0.42 | 0.034 | 0.66 | 0.44, 0.96 | -0.12 | 0.637 | 0.87 | 0.51, 1.50 | 0.26 | 0.225 | 1.30 | 0.85, 2.01 |
|  | Beyond primary education | 0.07 | 0.766 | 1.07 | 0.66, 1.73 | -0.54 | 0.143 | 0.57 | 0.27, 1.19 | 0.11 | 0.660 | 1.11 | 0.67, 1.85 |
|  | Exposure to tobacco smoke | -0.38 | 0.017 | 0.67 | 0.49, 0.93 |  |  |  |  | 0.52 | 0.003 | 1.68 | 1.19, 2.38 |
|  | Food secure | 0.04 | 0.781 | 1.05 | 0.74, 1.48 | 0.54 | 0.048 | 1.73 | 1.01, 3.04 | 0.33 | 0.079 | 1.38 | 0.96, 2.01 |
|  | Bazar food | -0.14 | 0.380 | 0.86 | 0.62, 1.19 | 0.26 | 0.286 | 1.30 | 0.81, 2.13 | -0.03 | 0.824 | 0.96 | 0.68, 1.35 |

**Table S11.** Full logistic regression models evaluating the association between betel quid use (binary yes/no) and anemia, diabetes, and hypertension for women. The beta coefficients for the logistic regression models represent log odds.

| **Men** | | **Anemia** | | | | **Diabetes** | | | | **Hypertension** | | | |
| --- | --- | --- | --- | --- | --- | --- | --- | --- | --- | --- | --- | --- | --- |
|  |  | **β** | **P** | **OR** | **CI** | **β** | **P** | **OR** | **CI** | **β** | **P** | **OR** | **CI** |
| **Bivariable analyses** | Betel nut use | 1.11 | 1.12e^-7^ | 3.04 | 2.02, 4.62 | -0.69 | 0.006 | 0.50 | 0.30, 0.81 | 0.01 | 0.962 | 1.01 | 0.68, 1.49 |
|  | | | | | | | | | | | | | |
| **Multivariable**  **analyses** | Betel nut use | 0.75 | 0.001 | 2.12 | 1.33, 3.37 | -0.84 | 0.003 | 0.43 | 0.24, 0.75 | -0.11 | 0.615 | 0.88 | 0.55, 1.41 |
|  | Age | 0.04 | 4.82e^-6^ | 1.05 | 1.02, 1.07 | 0.02 | 0.026 | 1.02 | 1.00, 1.05 | 0.03 | 0.001 | 1.03 | 1.02, 1.06 |
|  | MacArthur Ladder Present | -0.02 | 0.828 | 0.98 | 0.79, 1.19 | 0.33 | 0.006 | 1.40 | 1.10, 1.79 | 0.14 | 0.170 | 1.15 | 0.94, 1.41 |
|  | Primary education | -0.26 | 0.346 | 0.76 | 0.44, 1.32 | -0.10 | 0.772 | 0.90 | 0.45, 1.82 | 0.06 | 0.820 | 1.06 | 0.62, 1.84 |
|  | Beyond primary education | -0.53 | 0.076 | 0.58 | 0.32, 1.05 | -0.13 | 0.710 | 0.87 | 0.44,1.76 | 0.25 | 0.392 | 1.28 | 0.72, 2.31 |
|  | Tobacco use | -0.52 | 0.023 | 0.59 | 0.37, 0.92 |  |  |  |  | -0.27 | 0.212 | 0.75 | 0.48, 1.17 |
|  | Food secure | 0.11 | 0.644 | 1.12 | 0.68, 1.84 | 0.62 | 0.061 | 1.86 | 0.98, 3.68 | 0.15 | 0.544 | 1.16 | 0.71, 1.90 |
|  | Bazar food | -0.13 | 0.549 | 0.87 | 0.55, 1.36 | 0.66 | 0.014 | 1.95 | 1.14, 3.35 | 0.36 | 0.123 | 1.44 | 0.90, 2.30 |
|  | Laborer |  |  |  |  |  |  |  |  | -0.47 | 0.058 | 0.62 | 0.38, 1.01 |

**Table S12.** Full logistic regression models evaluating the association between betel quid use (binary yes/no) and anemia, diabetes, and hypertension for men. The beta coefficients for the logistic regression models represent log odds.

| **Women** | | **Anemia** | | | | **Diabetes** | | | | **Hypertension** | | | |
| --- | --- | --- | --- | --- | --- | --- | --- | --- | --- | --- | --- | --- | --- |
|  |  | **β** | **P** | **OR** | **CI** | **β** | **P** | **OR** | **CI** | **β** | **P** | **OR** | **CI** |
| **Bivariable analyses** | Infrequent | 0.01 | 0.972 | 1.01 | 0.49, 2.03 | -0.21 | 0.743 | 0.81 | 0.18, 2.43 | 0.72 | 0.056 | 2.06 | 1.00, 4.51 |
|  | Daily low | 0.41 | 0.027 | 1.50 | 1.04, 2.16 | 0.51 | 0.057 | 1.66 | 0.98, 2.82 | 0.76 | 8.25e^-5^ | 2.14 | 1.47, 3.14 |
|  | Daily high | 0.65 | 0.001 | 1.92 | 1.30, 2.86 | 0.46 | 0.107 | 1.59 | 0.89, 2.81 | 0.90 | 2.42e^-5^ | 2.47 | 1.63, 3.78 |
|  | | | | | | | | | | | | | |
| **Multivariable**  **analyses** | Infrequent | -0.22 | 0.555 | 0.80 | 0.37, 1.66 | -0.71 | 0.272 | 0.49 | 0.11, 1.53 | 0.13 | 0.739 | 1.14 | 0.52, 2.59 |
|  | Daily low | 0.17 | 0.438 | 1.18 | 0.76, 1.84 | -0.17 | 0.586 | 0.84 | 0.44, 1.57 | -0.02 | 0.923 | 0.97 | 0.61, 1.56 |
|  | Daily high | 0.39 | 0.088 | 1.49 | 0.94, 2.36 | -0.16 | 0.625 | 0.85 | 0.42, 1.64 | 0.16 | 0.514 | 1.17 | 0.72, 1.94 |
|  | Age | 0.02 | 0.008 | 1.02 | 1.01, 1.04 | 0.03 | 0.006 | 1.03 | 1.01, 1.06 | 0.05 | 2.13e^-9^ | 1.06 | 1.04, 1.08 |
|  | MacArthur Ladder Present | -0.10 | 0.024 | 0.90 | 0.82, 0.98 | 0.11 | 0.084 | 1.12 | 0.98, 1.27 | -0.02 | 0.651 | 0.97 | 0.89, 1.07 |
|  | Primary education | -0.47 | 0.018 | 0.62 | 0.42, 0.92 | -0.23 | 0.406 | 0.79 | 0.45, 1.36 | 0.24 | 0.275 | 1.27 | 0.82, 1.97 |
|  | Beyond primary education | 0.05 | 0.813 | 1.05 | 0.65, 1.71 | -0.57 | 0.126 | 0.56 | 0.26, 1.16 | 0.07 | 0.775 | 1.07 | 0.64, 1.79 |
|  | Exposure to tobacco smoke | -0.39 | 0.017 | 0.67 | 0.48, 0.93 |  |  |  |  | 0.51 | 0.003 | 1.66 | 1.18, 2.36 |
|  | Food secure | 0.07 | 0.674 | 1.07 | 0.75, 1.53 | 0.48 | 0.082 | 1.63 | 0.95, 2.88 | 0.36 | 0.055 | 1.43 | 0.99, 2.08 |
|  | Bazar food | -0.12 | 0.448 | 0.88 | 0.63, 1.21 | 0.18 | 0.460 | 1.20 | 0.74, 1.98 | -0.02 | 0.911 | 0.98 | 0.69, 1.38 |

**Table S13.** Full logistic regression models evaluating the association between frequency of betel quid use (categorical) and anemia, diabetes, and hypertension for women. The beta coefficients for the logistic regression models represent log odds.

| **Men** | | **Anemia** | | | | **Diabetes** | | | | **Hypertension** | | | |
| --- | --- | --- | --- | --- | --- | --- | --- | --- | --- | --- | --- | --- | --- |
|  |  | **β** | **P** | **OR** | **CI** | **β** | **P** | **OR** | **CI** | **β** | **P** | **OR** | **CI** |
| **Bivariable analyses** | Daily low | 1.52 | 1.66e^-7^ | 4.58 | 2.61, 8.19 | -1.08 | 0.007 | 0.33 | 0.14, 0.71 | 0.31 | 0.266 | 1.35 | 0.79, 2.33 |
|  | Daily high | 1.12 | 1.59e^-6^ | 3.07 | 1.95, 4.89 | -0.74 | 0.010 | 0.47 | 0.26, 0.82 | -0.18 | 0.421 | 0.83 | 0.53, 1.29 |
|  | | | | | | | | | | | | | |
| **Multivariable**  **analyses** | Daily low | 1.23 | 0.0001 | 3.43 | 1.82, 6.58 | -1.38 | 0.002 | 0.25 | 0.09, 0.57 | 0.29 | 0.361 | 1.34 | 0.71, 2.56 |
|  | Daily high | 0.72 | 0.005 | 2.06 | 1.23, 3.44 | -0.92 | 0.004 | 0.39 | 0.20, 0.74 | -0.34 | 0.198 | 0.71 | 0.42, 1.19 |
|  | Age | 0.04 | 3.01e^-5^ | 1.04 | 1.02, 1.06 | 0.03 | 0.009 | 1.03 | 1.01, 1.05 | 0.03 | 0.001 | 1.03 | 1.01, 1.05 |
|  | MacArthur Ladder Present | -0.01 | 0.957 | 0.99 | 0.81, 1.22 | 0.33 | 0.007 | 1.39 | 1.09, 1.79 | 0.14 | 0.149 | 1.16 | 0.94, 1.42 |
|  | Primary education | -0.25 | 0.357 | 0.77 | 0.44, 1.34 | -0.12 | 0.732 | 0.88 | 0.43, 1.79 | 0.07 | 0.800 | 1.07 | 0.62, 1.86 |
|  | Beyond primary education | -0.49 | 0.101 | 0.61 | 0.33, 1.10 | -0.20 | 0.569 | 0.82 | 0.40, 1.65 | 0.25 | 0.383 | 1.29 | 0.72, 2.32 |
|  | Tobacco use | -0.61 | 0.009 | 0.53 | 0.33, 0.85 |  |  |  |  | -0.35 | 0.117 | 0.69 | 0.44, 1.09 |
|  | Food secure | 0.11 | 0.660 | 1.12 | 0.67, 1.84 | 0.62 | 0.063 | 1.86 | 0.98, 3.71 | 0.12 | 0.629 | 1.12 | 0.69, 1.84 |
|  | Bazar food | -0.13 | 0.574 | 0.87 | 0.55, 1.38 | 0.65 | 0.016 | 1.92 | 1.13, 3.32 | 0.35 | 0.137 | 1.42 | 0.89, 2.28 |
|  | Laborer |  |  |  |  |  |  |  |  | -0.49 | 0.051 | 0.61 | 0.37, 1.01 |

**Table S14.** Full logistic regression models evaluating the association between frequency of betel quid use (categorical) and anemia, diabetes, and hypertension for men. The beta coefficients for the logistic regression models represent log odds.

| **Women** | | **CRP continuous** | | | | **CRP binary** | | | | **CRP ordinal** | | | |
| --- | --- | --- | --- | --- | --- | --- | --- | --- | --- | --- | --- | --- | --- |
|  |  | **β** | **P** | **OR** | **CI** | **β** | **P** | **OR** | **CI** | **β** | **P** | **OR** | **CI** |
| **Bivariable analyses** | Betel nut use | -0.09 | 0.213 | 0.90 | 0.78, 1.05- | -0.32 | 0.067 | 0.72 | 0.51, 1.02 | -0.32 | 0.071 | 0.72 | 0.51, 1.02 |
|  | | | | | | | | | | | | | |
| **Multivariable**  **analyses** | Betel nut use | -0.16 | 0.087 | 0.85 | 0.71, 1.02 | -0.48 | 0.029 | 0.61 | 0.39, 0.95 | -0.48 | 0.031 | 0.61 | 0.39, 0.95 |
|  | Age | 0.01 | 0.194 | 1.01 | 0.99, 1.01 | 0.01 | 0.309 | 1.00 | 0.99, 1.02 | 0.01 | 0.311 | 1.01 | 0.99, 1.02 |
|  | MacArthur Ladder Present | 0.03 | 0.112 | 1.03 | 0.99, 1.07 | 0.10 | 0.037 | 1.10 | 1.01, 1.22 | 0.10 | 0.037 | 1.11 | 1.01, 1.22 |
|  | Exposure to tobacco smoke | 0.10 | 0.193 | 1.10 | 0.95, 1.29 | 0.62 | 0.001 | 1.86 | 1.29, 2.69 | 0.62 | 0.001 | 1.85 | 1.28, 2.68 |
|  | Bazar food | 0.20 | 0.011 | 1.22 | 1.04, 1.42 | 0.32 | 0.100 | 1.37 | 0.94, 2.02 | 0.32 | 0.098 | 1.37 | 0.94 2.01 |
|  | Toilet owned or shared | -0.04 | 0.684 | 0.96 | 0.79, 1.16 | 0.04 | 0.849 | 1.04 | 0.65, 1.63 | 0.04 | 0.854 | 1.04 | 0.66, 1.64 |

**Table S15.** Full generalized linear model, logistic regression model, and ordinal logistic regression model evaluating the association between betel quid use (binary yes/no) and CRP as a continuous, binary, and ordinal variable for women. The beta coefficients for the logistic regression models represent log odds.

| **Men** | | **CRP continuous** | | | | **CRP binary** | | | | **CRP ordinal** | | | |
| --- | --- | --- | --- | --- | --- | --- | --- | --- | --- | --- | --- | --- | --- |
|  |  | **β** | **P** | **OR** | **CI** | **β** | **P** | **OR** | **CI** | **β** | **P** | **OR** | **CI** |
| **Bivariable analyses** | Betel nut use | -0.09 | 0.328 | 0.91 | 0.75, 1.09 | -0.25 | 0.324 | 0.77 | 0.47, 1.28 | -0.25 | 0.324 | 0.77 | 0.47, 1.28 |
|  | | | | | | | | | | | | | |
| **Multivariable**  **analyses** | Betel nut use | -0.17 | 0.097 | 0.84 | 0.68, 1.03 | -0.16 | 0.554 | 0.84 | 0.48, 1.47 | -0.16 | 0.553 | 0.84 | 0.48, 1.47 |
|  | Age | 0.01 | 0.012 | 1.01 | 1.00, 1.02 | -0.01 | 0.377 | 0.98 | 0.96, 1.01 | -0.01 | 0.377 | 0.98 | 0.96, 1.01 |
|  | MacArthur Ladder Present | 0.02 | 0.493 | 1.02 | 0.94, 1.12 | 0.10 | 0.367 | 1.11 | 0.88, 1.39 | 0.10 | 0.366 | 1.10 | 0.88, 1.39 |
|  | Tobacco use | 0.04 | 0.657 | 1.04 | 0.86, 1.27 | 0.24 | 0.348 | 1.28 | 0.75, 2.15 | 0.24 | 0.348 | 1.28 | 0.76, 2.15 |
|  | Bazar food | 0.02 | 0.831 | 1.02 | 0.84, 1.23 | 0.15 | 0.569 | 1.16 | 0.69, 1.96 | 0.15 | 0.568 | 1.16 | 0.69, 1.95 |
|  | Toilet owned or shared | -0.35 | 0.004 | 0.70 | 0.55, 0.89 | -0.54 | 0.145 | 0.58 | 0.26, 1.16 | -0.54 | 0.145 | 0.57 | 0.27, 1.21 |

**Table S16.** Full generalized linear model, logistic regression model, and ordinal logistic regression model evaluating the association between betel quid use (binary yes/no) and CRP as a continuous, binary, and ordinal variable for men. The beta coefficients for the logistic regression models represent log odds.

| **Women** | | **CRP continuous** | | | | **CRP binary** | | | | **CRP ordinal** | | | |
| --- | --- | --- | --- | --- | --- | --- | --- | --- | --- | --- | --- | --- | --- |
|  |  | **β** | **P** | **OR** | **CI** | **β** | **P** | **OR** | **CI** | **β** | **P** | **OR** | **CI** |
| **Bivariable analyses** | Infrequent | 0.17 | 0.339 | 1.18 | 0.83, 1.68 | -0.04 | 0.017 | 0.95 | 0.41, 2.07 | -0.04 | 0.917 | 0.95 | 0.43, 2.13 |
|  | Daily low | -0.13 | 0.152 | 0.87 | 0.73, 1.05 | -0.54 | 0.018 | 0.58 | 0.36, 0.91 | -0.54 | 0.018 | 0.58 | 0.37, 0.91 |
|  | Daily high | -0.16 | 0.109 | 0.85 | 0.70, 1.03 | -0.21 | 0.360 | 0.81 | 0.50, 1.26 | -0.21 | 0.382 | 0.81 | 0.51, 1.29 |
|  | | | | | | | | | | | | | |
| **Multivariable**  **analyses** | Infrequent | 0.10 | 0.574 | 1.11 | 0.77, 1.59 | -0.18 | 0.669 | 0.83 | 0.34, 1.87 | -0.18 | 0.671 | 0.83 | 0.36, 1.92 |
|  | Daily low | -0.21 | 0.046 | 0.81 | 0.65, 0.99 | -0.78 | 0.004 | 0.45 | 0.26, 0.77 | -0.77 | 0.004 | 0.45 | 0.26, 0.78 |
|  | Daily high | -0.21 | 0.066 | 0.81 | 0.64, 1.01 | -0.37 | 0.170 | 0.68 | 0.39, 1.16 | -0.36 | 0.183 | 0.69 | 0.49, 1.18 |
|  | Age | 0.01 | 0.175 | 1.01 | 0.99, 1.01 | 0.01 | 0.216 | 1.01 | 0.99, 1.03 | 0.01 | 0.218 | 1.01 | 0.99, 1.03 |
|  | MacArthur Ladder Present | 0.03 | 0.103 | 1.03 | 0.99, 1.08 | 0.11 | 0.029 | 1.12 | 1.01, 1.23 | 0.11 | 0.030 | 1.11 | 1.01, 1.23 |
|  | Exposure to tobacco smoke | 0.12 | 0,135 | 1.12 | 0.96, 1.32 | 0.66 | 0.0004 | 1.94 | 1.34, 2.82 | 0.66 | 0.001 | 1.94 | 1.33, 2.81 |
|  | Bazar food | 0.20 | 0.014 | 1.22 | 1.04, 1.42 | 0.31 | 0.116 | 1.36 | 0.93, 2.02 | 0.31 | 0.136 | 1.36 | 0.93, 2.01 |
|  | Toilet owned or shared | -0.02 | 0.856 | 0.98 | 0.81, 1.19 | 0.08 | 0.718 | 1.09 | 0.68, 1.71 | 0.08 | 0.725 | 1.08 | 0.68, 1.72 |

**Table S17.** Full generalized linear model, logistic regression model, and ordinal logistic regression model evaluating the association between frequency of betel quid use (categorical) and CRP as a continuous, binary, and ordinal variable for women. The beta coefficients for the logistic regression models represent log odds.

**Table S18.** Full generalized linear model, logistic regression model, and ordinal logistic regression model evaluating the association between frequency of betel quid use (categorical) and CRP as a continuous, binary, and ordinal variable for men. The beta coefficients for the logistic regression models represent log odds.

| **Men** | | **CRP continuous** | | | | **CRP binary** | | | | **CRP ordinal** | | | |
| --- | --- | --- | --- | --- | --- | --- | --- | --- | --- | --- | --- | --- | --- |
|  |  | **β** | **P** | **OR** | **CI** | **β** | **P** | **OR** | **CI** | **β** | **P** | **OR** | **CI** |
| **Bivariable analyses** | Daily low | 0.12 | 0.345 | 1.13 | 0.87, 1.46 | 0.17 | 0.579 | 1.19 | 0.62, 1.23 | 0.17 | 0.579 | 1.19 | 0.63, 2.25 |
|  | Daily high | -0.23 | 0.030 | 0.79 | 0.63, 0.97 | -0.62 | 0.046 | 0.53 | 0.28, 0.97 | -0.62 | 0.045 | 0.53 | 0.28, 0.98 |
|  | | | | | | | | | | | | | |
| **Multivariable  analyses** | Daily low | 0.02 | 0.882 | 1.02 | 0.77, 1.34 | 0.23 | 0.519 | 1.26 | 0.61, 2.58 | 0.23 | 0.52 | 1.26 | 0.61, 2.59 |
|  | Daily high | -0.30 | 0.007 | 0.73 | 0.58, 0.92 | -0.48 | 0.148 | 0.61 | 0.31, 1.17 | -0.48 | 0.14 | 0.61 | 0.31, 1.18 |
|  | Age | 0.01 | 0.014 | 1.01 | 1.00, 1.02 | -0.01 | 0.357 | 0.98 | 0.96, 1.01 | -0.01 | 0.35 | 0.98 | 0.96, 1.01 |
|  | MacArthur Ladder Present | 0.02 | 0.525 | 1.02 | 0.84, 1.11 | 0.10 | 0.374 | 1.10 | 0.88, 1.39 | 0.10 | 0.37 | 1.10 | 0.88, 1.39 |
|  | Tobacco use | 0.0003 | 0.996 | 1.00 | 0.82, 1.33 | 0.16 | 0.552 | 1.17 | 0.68, 1.99 | 0.16 | 0.55 | 1.17 | 0.68, 2.00 |
|  | Bazar food | 0.01 | 0.896 | 1.01 | 0.83, 1.22 | 0.14 | 0.605 | 1.14 | 0.68, 1.93 | 0.13 | 0.61 | 1.14 | 0.68, 1.92 |
|  | Toilet owned or shared | -0.35 | 0.003 | 0.69 | 0.54, 0.89 | -0.55 | 0.138 | 0.57 | 0.26, 1.15 | -0.55 | 0.13 | 0.57 | 0.28 |

**Table S19.** Counts for multiple betel categories.

|  | **1** | **2** | **3** | **4** | **5** |
| --- | --- | --- | --- | --- | --- |
| **Betel use** | No use + infrequent | 1-2/day | 3-4/day | 5-7/day | ≥8/day |
| **Count** | 498 | 130 | 166 | 131 | 140 |

**Table S20.** Full logistic regression models evaluating the association between frequency of betel quid use (categorical) and anemia, diabetes, and hypertension for men and women. The beta coefficients for the logistic regression models represent log odds.

|  | | **Anemia** | | | | **Diabetes** | | | | **Hypertension** | | | |
| --- | --- | --- | --- | --- | --- | --- | --- | --- | --- | --- | --- | --- | --- |
|  |  | **β** | **P** | **OR** | **CI** | **β** | **P** | **OR** | **CI** | **β** | **P** | **OR** | **CI** |
| **Bivariable analyses** | 1-2/day | 0.39 | 0.102 | 1.47 | 0.92, 2.36 | 0.04 | 0.876 | 1.04 | 0.57, 1.91 | 1.24 | 7.33e^-6^ | 3.46 | 2.01, 5.97 |
|  | 3-4/day | 0.99 | 2.36e^-7^ | 2.69 | 1.85, 3.93 | -0.19 | 0.449 | 0.82 | 0.50, 1.35 | 0.32 | 0.084 | 1.38 | 0.95, 1.99 |
|  | 5-7/day | 0.89 | 1.35e^-5^ | 2.45 | 1.63, 3.67 | -0.10 | 0.707 | 0.90 | 0.53, 1.53 | 0.50 | 0.015 | 1.65 | 1.10, 2.48 |
|  | ≥8/day | 0.74 | 0.0001 | 2.11 | 1.43, 3.12 | -0.02 | 0.914 | 0.97 | 0.59, 1.60 | 0.09 | 0.626 | 1.10 | 0.74, 1.62 |
|  | | | | | | | | | | | | | |
| **Multivariable**  **analyses** | 1-2/day | 0.45 | 0.528 | 1.57 | 0.38, 6.45 | -0.51 | 0.539 | 0.59 | 0.11, 3.13 | 1.19 | 0.174 | 3.29 | 0.58, 18.4 |
|  | 3-4/day | 1.56 | 3.75e^-6^ | 4.77 | 2.46, 9.26 | -1.62 | 0.001 | 0.19 | 0.07, 0.50 | -0.44 | 0.185 | 0.64 | 0.33, 1.23 |
|  | 5-7/day | 0.79 | 0.017 | 2.21 | 1.15, 4.26 | -1.23 | 0.006 | 0.29 | 0.11, 0.70 | -0.63 | 0.070 | 0.53 | 0.26, 1.05 |
|  | ≥8/day | 1.01 | 0.001 | 2.74 | 1.52, 4.94 | -0.86 | 0.017 | 0.42 | 0.20, 0.85 | -0.68 | 0.028 | 0.50 | 0.27, 0.93 |
|  | Women | 0.98 | 3.81e^-6^ | 2.68 | 1.76, 4.08 | -1.11 | 2.01e^-5^ | 0.32 | 0.19, 0.54 | 0.85 | 6.01e^-5^ | 2.34 | 1.54, 3.56 |
|  | 1-2/day* Women | -0.61 | 0.422 | 0.54 | 0.12, 2.41 | 0.57 | 0.532 | 1.77 | 0.29, 10.6 | -0.61 | 0.504 | 0.53 | 0.08, 3.32 |
|  | 3-4/day* Women | -1.34 | 0.001 | 0.26 | 0.11, 0.57 | 1.61 | 0.004 | 5.02 | 1.64, 15.3 | 0.18 | 0.654 | 1.20 | 0.54, 2.67 |
|  | 5-7/day* Women | -0.34 | 0.413 | 0.70 | 0.31, 1.62 | 1.36 | 0.017 | 3.92 | 1.27, 12.1 | 1.15 | 0.012 | 3.17 | 1.28, 7.85 |
|  | ≥8/day* Women | -0.86 | 0.033 | 0.42 | 0.18, 0.93 | 0.83 | 0.116 | 2.29 | 0.81, 6.50 | 0.75 | 0.083 | 2.13 | 0.91, 5.02 |
|  | Age | 0.03 | 8.75e^-6^ | 1.03 | 1.02, 1.04 | 0.04 | 5.36e^-5^ | 1.03 | 1.02, 1.05 | 0.06 | 7.61e^-14^ | 1.06 | 1.04, 1.08 |
|  | MacArthur Ladder Present | -0.09 | 0.030 | 0.91 | 0.83, 0.99 | 0.16 | 0.005 | 1.17 | 1.05, 1.31 | 0.00 | 0.995 | 1.00 | 0.91, 1.10 |
|  | Primary education | -0.40 | 0.016 | 0.66 | 0.48, 0.93 | -0.17 | 0.416 | 0.83 | 0.46, 1.21 | 0.25 | 0.160 | 1.29 | 0.90, 1.84 |
|  | Beyond primary education | -0.07 | 0.677 | 0.92 | 0.63, 1.33 | -0.28 | 0.242 | 0.75 | 0.46, 1.21 | 0.29 | 0.139 | 1.34 | 0.91, 1.99 |
|  | Exposure to tobacco smoke | -0.48 | 0.0004 | 0.61 | 0.46, 0.81 |  |  |  |  | 0.21 | 0.143 | 1.24 | 0.93, 1.65 |
|  | Food secure | 0.07 | 0.639 | 1.07 | 0.79, 1.44 | 0.55 | 0.009 | 1.74 | 1.13, 2.66 | 0.37 | 0.023 | 1.44 | 1.05, 1.99 |
|  | Bazar food | -0.15 | 0.262 | 0.85 | 0.65, 1.12 | 0.38 | 0.039 | 1.47 | 1.02, 2.10 | 0.12 | 0.376 | 1.13 | 0.85,1.51 |

**Table S21.** Full logistic regression models evaluating the association between frequency of betel quid use (5 levels, ordinal) and anemia, diabetes, and hypertension for men and women. The beta coefficients for the logistic regression models represent log odds.

|  | | **Anemia** | | | | **Diabetes** | | | | **Hypertension** | | | |
| --- | --- | --- | --- | --- | --- | --- | --- | --- | --- | --- | --- | --- | --- |
|  |  | **β** | **P** | **OR** | **CI** | **β** | **P** | **OR** | **CI** | **β** | **P** | **OR** | **CI** |
| **Bivariable analyses** | Betel nut use | 0.24 | 2.27e^-8^ | 1.27 | 1.17, 1.39 | -0.02 | 0.676 | 0.97 | 0.87, 1.08 | 0.06 | 0.132 | 1.06 | 0.98, 1.16 |
|  | | | | | | | | | | | | | |
| **Multivariable**  **analyses** | Betel nut use | 0.27 | 7.69e^-5^ | 1.31 | 1.14, 1.50 | -0.30 | 0.001 | 0.73 | 0.62, 0.88 | -0.18 | 0.009 | 0.82 | 0.71, 0.95 |
|  | Women | 1.05 | 7.96e^-5^ | 2.86 | 1.69, 4.82 | -1.33 | 5.70e^-5^ | 0.26 | 0.13, 0.50 | 0.64 | 0.017 | 1.90 | 1.12, 3.22 |
|  | Betel nut use*Women | -0.19 | 0.025 | 0.82 | 0.68, 0.97 | 0.32 | 0.006 | 1.37 | 1.09, 1.73 | 0.23 | 0.014 | 1.26 | 1.04, 1.51 |
|  | Age | 0.03 | 1.58e^-6^ | 1.03 | 1.01, 1.04 | 0.03 | 0.0001 | 1.03 | 1.01, 1.05 | 0.06 | 8.28e^-15^ | 1.06 | 1.03, 1.07 |
|  | MacArthur Ladder Present | -0.10 | 0.017 | 0.90 | 0.82, 0.98 | 0.15 | 0.005 | 1.17 | 1.04, 1.31 | 0.002 | 0.957 | 1.00 | 0.91, 1.09 |
|  | Primary education | -0.39 | 0.017 | 0.67 | 0.48, 0.93 | -0.16 | 0.456 | 0.85 | 0.55, 1.30 | 0.24 | 0.172 | 1.28 | 0.89, 1.82 |
|  | Beyond primary education | -0.08 | 0.659 | 0.92 | 0.63, 1.32 | -0.26 | 0.280 | 0.77 | 0.47, 1.23 | 0.28 | 0.153 | 1.33 | 0.90, 1.97 |
|  | Exposure to tobacco smoke | -0.42 | 0.002 | 0.66 | 0.50, 0.86 |  |  |  |  | 0.22 | 0.123 | 1.25 | 0.94, 1.67 |
|  | Food secure | 0.11 | 0.484 | 1.11 | 0.82, 1.49 | 0.54 | 0.010 | 1.73 | 1.13, 2.63 | 0.34 | 0.035 | 1.40 | 1.02, 1.93 |
|  | Bazar food | -0.16 | 0.219 | 0.84 | 0.64, 1.11 | 0.41 | 0.024 | 1.51 | 1.09, 1.73 | 0.13 | 0.387 | 1.13 | 0.85, 1.51 |

**Table S22.** Full generalized linear model, logistic regression model, and ordinal logistic regression model evaluating the association between frequency of betel quid use (categorical) and CRP as a continuous, binary, and ordinal variable for men and women. The beta coefficients for the logistic regression models represent log odds.

|  | | **CRP continuous** | | | | **CRP binary** | | | | **CRP ordinal** | | | |
| --- | --- | --- | --- | --- | --- | --- | --- | --- | --- | --- | --- | --- | --- |
|  |  | **β** | **P** | **OR** | **CI** | **β** | **P** | **OR** | **CI** | **β** | **P** | **OR** | **CI** |
| **Bivariable analyses** | 1-2/day | -0.19 | 0.087 | 0.82 | 0.66, 1.02 | -0.88 | 0.008 | 0.41 | 0.21, 0.80 | -0.88 | 0.008 | 0.41 | 0.21, 0.80 |
|  | 3-4/day | 0.01 | 0.884 | 1.01 | 0.85, 1.20 | -0.07 | 0.731 | 0.93 | 0.62, 1.39 | -0.07 | 0.731 | 0.93 | 0.62, 1.39 |
|  | 5-7/day | -0.18 | 0.058 | 0.83 | 0.69, 1.01 | -0.31 | 0.183 | 0.73 | 0.45, 1.16 | -0.31 | 0.184 | 0.72 | 0.45, 1.16 |
|  | ≥8/day | -0.26 | 0.004 | 0.76 | 0.63, 0.92 | -0.50 | 0.041 | 0.60 | 0.37, 0.98 | -0.48 | 0.045 | 0.61 | 0.37, 0.99 |
|  | | | | | | | | | | | | | |
| **Multivariable**  **analyses** | 1-2/day | -0.33 | 0.004 | 0.72 | 0.57, 0.90 | -1.04 | 0.003 | 0.35 | 0.17, 0.70 | -1.04 | 0.003 | 0.35 | 0.17, 0.70 |
|  | 3-4/day | -0.97 | 0.302 | 0.91 | 0.75, 1.09 | -0.20 | 0.377 | 0.81 | 0.51, 1.28 | -0.20 | 0.379 | 0.81 | 0.51, 1.28 |
|  | 5-7/day | -0.23 | 0.019 | 0.78 | 0.64, 0.96 | -0.31 | 0.231 | 0.73 | 0.44, 1.21 | -0.31 | 0.233 | 0.73 | 0.44, 1.22 |
|  | ≥8/day | -0.29 | 0.002 | 0.74 | 0.61, 0.90 | -0.46 | 0.079 | 0.63 | 0.37, 1.06 | -0.44 | 0.087 | 0.63 | 0.38, 1.06 |
|  | Women | 0.14 | 0.025 | 1.16 | 1.02, 1.32 | 0.23 | 0.177 | 1.26 | 0.90, 1.77 | 0.23 | 0.174 | 1.26 | 0.90, 1.77 |
|  | Age | 0.01 | 0.005 | 1.01 | 1.00,1.01 | 0.003 | 0.629 | 1.00 | 0.98, 1.02 | 0.003 | 0.629 | 1.00 | 0.98, 1.02 |
|  | MacArthur Ladder Present | 0.03 | 0.109 | 1.03 | 0.99, 1.07 | 0.11 | 0.020 | 1.11 | 1.02, 1.22 | 0.10 | 0.021 | 1.11 | 1.02, 1.21 |
|  | Exposure to tobacco smoke | 0.07 | 0.225 | 1.07 | 0.95, 1.22 | 0.51 | 0.001 | 1.67 | 1.24, 2.26 | 0.51 | 0.001 | 1.66 | 1.24, 2.25 |
|  | Bazar food | 0.12 | 0.057 | 1.12 | 0.99, 1.27 | 0.24 | 0.122 | 1.27 | 0.94, 1.73 | 0.24 | 0.120 | 1.27 | 0.94, 1.73 |
|  | Toilet owned or shared | -0.14 | 0.064 | 0.86 | 0.74, 1.01 | -0.08 | 0.676 | 0.92 | 0.63, 1.35 | -0.08 | 0.672 | 0.92 | 0.63, 1.34 |

**Table S23.** Full generalized linear model, logistic regression model, and ordinal logistic regression model evaluating the association between frequency of betel quid use (5 levels, ordinal) and CRP as a continuous, binary, and ordinal variable for men and women. The beta coefficients for the logistic regression models represent log odds.

|  | | **CRP continuous** | | | | **CRP binary** | | | | **CRP ordinal** | | | |
| --- | --- | --- | --- | --- | --- | --- | --- | --- | --- | --- | --- | --- | --- |
|  |  | **β** | **P** | **OR** | **CI** | **β** | **P** | **OR** | **CI** | **β** | **P** | **OR** | **CI** |
| **Bivariable analyses** | Betel nut use | -0.05 | 0.005 | 0.94 | 0.90, 0.98 | -0.10 | 0.039 | 0.90 | 0.82, 0.99 | -0.10 | 1.39e^-14^ | 0.90 | 0.81, 0.99 |
|  | | | | | | | | | | | | | |
| **Multivariable**  **analyses** | Betel nut use | -0.06 | 0.002 | 0.93 | 0.89, 0.97 | -0.09 | 0.100 | 0.90 | 0.81, 1.02 | -0.09 | 0.107 | 0.91 | 0.81, 1.02 |
|  | Women | 0.12 | 0.059 | 1.13 | 0.99, 1.28 | 0.14 | 0.384 | 1.15 | 0.83, 1.61 | 0.14 | 0.380 | 1.15 | 0.83, 1.61 |
|  | Age | 0.01 | 0.010 | 1.01 | 1.00, 1.01 | -0.0001 | 0.977 | 0.99 | 0.98, 1.01 | -0.0002 | 0.973 | 0.99 | 0.98, 1.01 |
|  | MacArthur Ladder Present | 0.03 | 0.125 | 1.02 | 0.99, 1.06 | 0.10 | 0.029 | 1.10 | 1.01, 1.21 | 0.10 | 0.029 | 1.10 | 1.01, 1.21 |
|  | Exposure to tobacco smoke | 0.08 | 0.196 | 1.08 | 0.95, 1.22 | 0.51 | 0.001 | 1.66 | 1.23, 2.24 | 0.51 | 0.001 | 1.66 | 1.23, 2.24 |
|  | Bazar food | 0.12 | 0.061 | 1.12 | 0.99, 1.26 | 0.24 | 0.120 | 1.27 | 0.93, 1.73 | 0.24 | 0.117 | 1.27 | 0.94, 1.73 |
|  | Toilet owned or shared | -0.14 | 0.052 | 0.86 | 0.74, 1.00 | -0.09 | 0.607 | 0.91 | 0.61, 1.32 | -0.10 | 0.604 | 0.90 | 0.62, 1.32 |
